# Determinants of Weight Gain Prevention in Young Adult and Midlife Women: Study Design and Protocol of a Randomized Controlled Trial

**DOI:** 10.2196/resprot.4008

**Published:** 2015-03-26

**Authors:** Catherine J Metzgar, Sharon M Nickols-Richardson

**Affiliations:** ^1^University of Illinois at Urbana-ChampaignDepartment of Food Science and Human NutritionUrbana, ILUnited States

**Keywords:** body weight, weight gain prevention, weight maintenance, women

## Abstract

**Background:**

Treatment of overweight and obesity through body weight reduction has been monumentally ineffective as few individuals are able to sustain weight loss. Rather than treating weight gain once it has become problematic, prevention of weight gain over time may be more effective.

**Objective:**

The aim of this research is to preclude the burden of adult obesity in women by identifying the determinants of weight gain prevention. The objective of this randomized controlled trial (RCT) is to compare a weight gain prevention intervention delivered by the registered dietitian versus counselor.

**Methods:**

This is a 12-month parallel-arm weight gain prevention RCT designed to increase self-efficacy, self-regulation, outcome expectations and family and social support through the use of a nutrition education intervention in women, aged 18-45 years, from the Urbana-Champaign (Illinois, USA) area. Women have been randomized to registered dietitian, counselor or wait-list control groups (August 2014) and are undergoing weekly nutrition education sessions for four months, followed by monthly sessions for eight months (through August 2015). Outcome measures, including: (1) dietary intake, (2) physical activity, (3) anthropometric and blood pressure measurements, (4) biochemical markers of health, (5) eating behaviors and health perceptions, and (6) mediators of behavior change, were collected before the intervention began (baseline) and will be collected at 3, 6, 9, and 12 months of the study.

**Results:**

In total, 87 women have been randomized to intervention groups, and 81 women have completed first week of the study. Results are expected in early 2016.

**Conclusions:**

This RCT is one of the first to examine weight gain prevention in women across normal, overweight, and obese body mass index categories. Results of this research are expected to have application to evidence-based practice in weight gain prevention for women and possibly have implication for policy regarding decreasing the encumbrance of overweight and obesity in the United States.

## Introduction

### Adult Weight Management

Small weight gains over time, around 1-2 pounds per year [1,2], contribute to the development of overweight and obesity. Once established, obesity is difficult to treat [3], as reduction of excess body weight is rarely effective in the long term. Short-term weight loss can be achieved by a variety of methods, but few of these approaches are sustainable and effective in facilitating permanent weight loss [4-10]. On average, individuals adhere to weight loss programs for approximately six months [11]; following weight loss, most individuals regain half of the weight lost within one year, and return to baseline weight within 3-5 years [11-13]. Weight gain prevention, on the other hand, avoids the difficulties that may accompany weight loss and its maintenance and offers an alternative option for weight management.

To reduce disease risk and improve overall health, effective weight gain prevention is essential; however, few interventions have successfully examined weight gain prevention and little is known about the determinants of and strategies for preventing weight gain over the long term. Much of the existing research has focused on treatment of overweight and obesity through reduction of excess body weight [14,15] or prevention of weight regain following weight loss [16-20].

### Weight Gain Prevention

In the first weight gain prevention trial, normal weight adults, aged 25-74 years, were randomized to an untreated control group or a treatment group that received monthly newsletters plus a financial incentive for weight maintenance for one year [21]. The treatment group experienced an average weight loss of 1 kg, which was significantly different from the control group; with the treatment effect being stronger in men than women [21]. Building upon the Pound of Prevention (POP) work, 3-year weight gain prevention in adults, aged 20-45 years, was investigated [22,23]. Participants were randomized to a no-contact control group or to one of two education groups that received nutrition education via monthly newsletters and semiannual nutrition and exercise classes. One education group received a lottery incentive for participation. Significant differences in weight gain between the control and education groups were not found, although weight-related behaviors did improve in participants receiving education [22,23].

The Shape Program was a medium-intensity behavioral intervention in overweight and class I obese premenopausal black women that included weekly self-monitoring, monthly counseling calls, tailored skills training and a YMCA gym membership and was compared to usual care that included newsletters covering general wellness topics every six months during the 18-month study [24]. After one year, weight loss was significantly greater in the intervention group; these changes were sustained at 18 months. No significant differences in waist circumference, blood pressure, glucose or lipid levels between the intervention and usual care groups were observed at any measurement point during the study [24]. Levine and colleagues [25] randomized normal weight and overweight women to a clinic-based group, a correspondence group or a control group for 24 months. During three years, the intervention had no influence on weight gain in either group; however, age, dieting status, and feelings of hunger were found to be predictive of weight gain.

The Groningen Overweight and Lifestyle (GOAL) study examined weight gain prevention in overweight and obese men and women with hypertension and/or dyslipidemia in the Netherlands by comparing the effects of lifestyle counseling by a nurse practitioner to usual care from a general practitioner during a 1-year period [26]. No significant differences were observed in weight change between groups at one year or after three years [26,27]. Study of Novel Approaches to Weight Gain Prevention (SNAP) is the most recently published intervention [28]. Two novel self-regulation approaches to weight gain prevention—small consistent changes and large periodic changes—were compared to a minimal treatment control for an average of three years of follow-up. Results of this study have not yet been published [28]. Without complete knowledge of the determinants of and strategies for weight gain prevention, public health will remain at risk for complications and costs related to overweight and obesity. Weight gain prevention offers a primary strategy for weight management and obesity prevention [24,26].

Women who previously participated in a weight-loss intervention identified gender-specific life transitions and stressors, including pregnancy, post pregnancy, family responsibilities, health status changes, and aging as precipitators of weight gain [29]. Young adulthood and perimenopause appear to be critical intervals for weight gain [30-32]; therefore, weight gain prevention efforts should target these lifespan stages, specifically in women.

### Aims and Objectives

The current study aims to identify determinants of weight gain prevention in young adult and midlife premenopausal women through a 1-year weight gain prevention intervention that includes nutrition education. We hypothesize that compared to a wait-list control group, women who participate in a weight gain prevention intervention designed to increase self-efficacy, self-regulation, outcome expectations, and family and social support will maintain current body weight during a 12-month period. It is further hypothesized that women in an intervention group led by registered dietitians will have less weight gain during 12 months compared to women in an intervention group led by counselors.

## Methods

### Recruitment, Screening, and Enrollment

**Figure 1:**
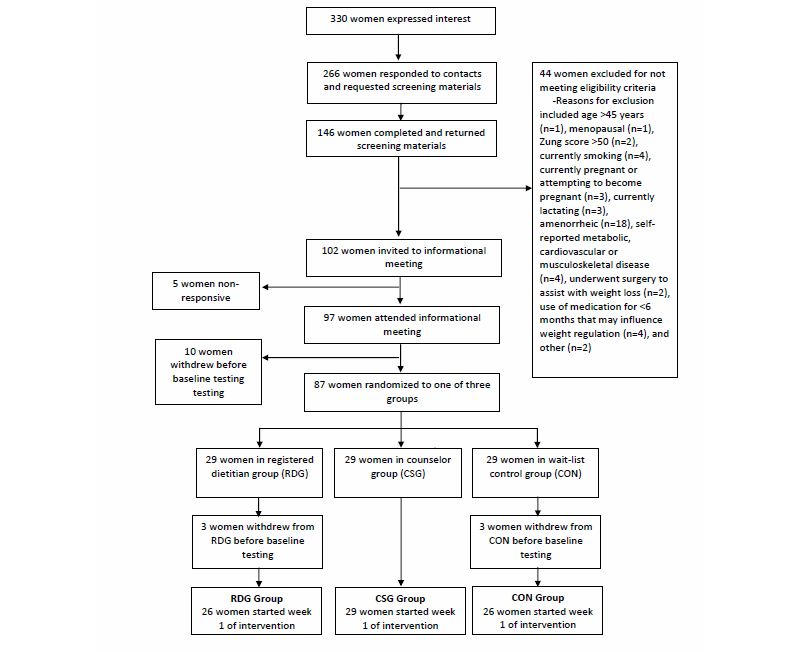
Diagram of recruitment, enrollment and randomization of participants in a study examining weight gain prevention in young adult and midlife women.

Participants were recruited by word-of-mouth, electronic mail messages, and posted flyers from the University of Illinois campus and the Urbana-Champaign (IL, USA) communities. A flow diagram of response, screening, and randomization steps is displayed in Figure 1. A total of 330 women responded to recruitment methods, between June and August 2014. Of these, 266 women met prescreening criteria (appropriate age, body mass index [BMI], and desire to prevent weight gain) and received screening materials including a medical history form, Zung Self-Rating Depression Scale/Status Inventory [33], and informed consent. A total of 146 women returned screening materials, which were reviewed by investigators. One hundred two women met eligibility criteria for participation, and 87 women were randomized, with 81 women completing baseline testing.

The current study included premenopausal women between the ages of 18-45 years with a BMI of >18.5 kg/m^2^. There were no additional criteria for body weight and BMI to ensure participation by women from a range of weight status categories. Further inclusion criteria included eumenorrhea (≥8 menstrual cycles/year), score of <50 on the Zung Self-Rating Depression Scale/Status Inventory [33], and no self-reported metabolic, cardiovascular or musculoskeletal diseases or use of medications or supplements to manage a chronic health condition. Exclusion criteria included women who currently smoked, were pregnant or attempting to become pregnant or were currently lactating. Women using medications influencing weight regulation, such as steroid or thyroid hormones or oral contraceptives, were excluded if use was for <2 months before the start of the study. Gastric bypass surgery was also an exclusion criterion.

The Institutional Review Board (IRB) for the protection of human subjects at the University of Illinois at Urbana-Champaign (UIUC) approved the study protocol (UIUC IRB#14397). Each participant provided written informed consent before study participation.

### Study Design

The current study is a 12-month parallel-arm weight gain prevention randomized controlled trial. After enrollment, women were randomized to one of three intervention groups: (1) weight gain prevention intervention delivered by a registered dietitian (RDG); (2) weight gain prevention intervention delivered by a counselor (CSG), or (3) wait-list control (CON) group. The RDG and CSG weight gain prevention interventions are identical in materials and content; the only difference is the credentialing of the individuals leading the intervention. Women in the CON group receive no intervention; upon completion of the 12-month waiting period, these women will be randomized to the RDG group or CSG group and will receive the respective intervention for the next 12-month period.

### Intervention

During the 1-year study, women randomized to the RDG and CSG groups will attend a total of 24, 1-hour nutrition education sessions that are based on effective weight-loss programs/plans which address energy balance through sustainable diet, exercise, and behavior modifications [30,34-39]. These sessions will be held weekly for 16 weeks (months 1-4) and monthly thereafter (months 5-12) [30]. Vegetable consumption, planning ahead for food intake and portion control will be emphasized [34,35], and general nutrition information, eating away from home, food selection, food preparation, and recipe modification also will be addressed [34-37]. Other topics will include fitness and physical activity, culinary skills, breakfast consumption, healthy snacking and beverage choices, nutrient density, family menu planning, and grocery shopping. Problem solving, motivational concerns, and stress management will be encouraged [30,34-39]. Education sessions will relay constructs of the Social Cognitive Theory (SCT) [40].

Education sessions will follow a three-part format. Each session will begin with a brief review of information covered in the previous session and will address participant progress, including successes, challenges and questions. Next, the leader will deliver the nutrition education component of the session using an interactive group discussion format. Participants will be provided with handouts addressing food choices, dietary patterns, menu plans, and other information pertaining to the lesson. Finally, the content for the lesson will be summarized and participants will have a chance to ask questions, address concerns and set specific behavioral goals for the next session. Education sessions will be randomly selected for evaluation by a process observer who will rate the sessions based on investigator-established criteria.

Four registered dietitians will deliver the intervention to women in the RDG group. All women in the RDG group will equally interact with all four registered dietitians during the study. Four counselors will deliver the intervention to women in the CSG group, with these women having equal interaction with all four counselors across the study. The credentials of the professionals delivering the intervention will not be revealed to participants until after completion of the study. The registered dietitians are all female and have been practicing for <5 years. The counselors are all female and are graduate teaching assistants at UIUC in programs unrelated to nutrition or dietetics. Compliance will be defined as attendance of >85% of education sessions. If women are unable to attend an education session, virtual make-up sessions will be offered, along with a quiz. Completion and return of the quiz will indicate that the materials were studied and reviewed and that the participant was compliant.

### Outcome Measures

Before the intervention (baseline), data on dietary intake, physical activity, anthropometric, and blood pressure measurements, biochemical markers of health, eating behaviors and health perceptions, and SCT mediators of behavioral change were collected. These outcome measures also will be obtained at 3, 6, 9 and 12 months.

### Dietary Intake and Physical Activity Assessment

Participants were taught to accurately complete 4-day food records and the Stanford 7-Day Physical Activity Recall Scale [41]. To ensure accuracy in recording foods and beverage consumption, handouts containing examples of standard serving sizes were provided. Participants recorded all food and beverages consumed for three non-consecutive weekdays and one weekend day before the baseline testing session [35]. Four-day food records will be analyzed using the Nutrition Data System for Research (NDSR) dietary analysis software (Nutrition Coordinating Center, Minneapolis, MN, USA) to estimate average daily dietary intake of total energy (kcal/day), carbohydrate (g/day), protein (g/day), fat (g/day), and fiber (g/day) in addition to intake by food groups (svgs/day).

For seven consecutive days before the baseline testing session, participants recorded the number of hours slept, spent in front of a television or computer screen, and engaged in moderate, hard, and very hard physical activity [35]. Participants wore accelerometers at the waist, wrist, or ankle during all waking hours for seven consecutive days while also recording physical activity to provide an objective assessment of energy expenditure. Approximately 70% of participants in each group wore accelerometers as they were not available for all individuals. Physical activity records will be analyzed by summing total hours of moderate, hard, and very hard activity and dividing by seven to estimate hours of physical activity per day. These records will be further analyzed by converting activities into metabolic equivalents (METs) (hr/d), which will be evaluated as light activity (1-3 METs), moderate activity (>3-6 METs), and vigorous activity (>6 METs) to estimate the number of calories expended per day. Accelerometry data will be analyzed using ActiLife 6.11 (ActiGraph, Pensacola, FL, USA) to estimate the number of calories expended per day, the MET rate per day, and the length of time (minutes) spent in sedentary, light, moderate, vigorous, and very vigorous activities.

### Anthropometric and Blood Pressure Measurements

Baseline standing height (cm) was recorded to the nearest 0.1 cm using a calibrated scale-mounted stadiometer (Seca 700, Hanover, MD, USA). Body weight (kg) was measured using a calibrated scale (Tanita 410GS, Arlington Heights, IL, USA) to the nearest 0.1 kg. BMI (kg/m^2^) was calculated using height and body weight measurements. A retractable measuring tape (Gulik II, Country Technology, Inc, Gay Mills, WI) was used to measure waist (cm) and hip (cm) circumferences, in duplicate, to the nearest 0.1 cm according to standard protocol [34]. Waist circumference was measured at the narrowest point of the waist, approximately one inch above the navel, and hip circumference was measured at the widest part of the buttocks [35]. Waist and hip circumference measurements were averaged to obtain a single value for each site; these values were used to calculate the waist:hip ratio. Fat mass (FM; kg) and body fat percentage (BF%) were measured using a Tanita scale (410GS).

Seated systolic and diastolic blood pressures (mm Hg) were measured by a trained study investigator using a standard sphygmomanometer (Baumanometer® Desk Model, Copiague, NY, USA) following a 5-minute rest period. Blood pressure measurements were taken in duplicate with a 2-3-minute rest period between readings; mean systolic arterial pressure values and diastolic arterial pressure values will be used in data analyses. Resting heart rate was also measured after the 5-minute rest period.

### Biochemical Markers of Health

Venous blood samples (~30 mL) were collected by a trained phlebotomist between 7:00 to 9:30 AM after a 12-hour fast. Whole blood samples were processed and stored at -80⁰C. Serum will be analyzed for concentrations of insulin, glucose, total cholesterol, high-density lipoprotein-cholesterol (HDL-C), low-density lipoprotein-cholesterol (LDL-C), triacylglycerides (TG), leptin, adiponectin, and resistin.

Serum insulin (µU/mL) (LINCO Research, St Charles, MO, USA) will be measured using enzyme-linked immunosorbent assay (ELISA), and serum glucose (mg/dL) (Stanbio Labs, Boerne, TX, USA) will be measured by spectrophotometry. Total cholesterol (mg/dL), HDL-C (mg/dL) and TG (mg/dL) concentrations will be measured by spectrophotometry using the total cholesterol, HDL-C and TG kits, respectively (Stanbio Labs). Total cholesterol, HDL-C and TG concentrations will be used to calculate LDL-C concentration (mg/dL) using the equation: LDL-C=total cholesterol - HDL-C - (TG/5) [42]. Serum leptin (ng/mL), adiponectin (ng/mL), and resistin (ng/mL) will be measured using ELISA (R&D Systems, Minneapolis, MN, USA). All serum samples for each biomarker will be analyzed in duplicate at corresponding study intervals. Intra- and inter-assay coefficients of variations (CV) are <15% for all kits.

### Eating Behaviors, Health Perceptions, and SCT Mediators of Behavioral Change

Participants completed questionnaires designed to evaluate eating behaviors, health perceptions, perseverance, and SCT mediators. The Eating Inventory [43] will evaluate ratings of cognitive eating restraint, hunger, and disinhibition. The Short-Form 36 Health Survey (SF-36) [44] will assess self-reported health issues. Perseverance will be examined using the Short Grit Scale (Grit-S) [45], and an investigator-designed questionnaire will evaluate SCT mediators, including self-efficacy, outcome expectations, self-regulation, and social and family support. Standard scoring and interpretation methods will be used to evaluate all questionnaires [43-45].

### Statistical Analysis

Baseline characteristics of study participants were characterized using descriptive statistics: mean (SD). Participants in the three intervention groups (Treatment) will participate in five data collection sessions at specified intervals (Time). The Shapiro-Wilk test for normality will be used to test for normality and homogeneity of variance within groups; data will be transformed if necessary. Body weight, BMI, waist : hip ratio, FM, BF%, systolic and diastolic blood pressure, serum insulin, glucose, TC, HDL-C, LDL-C, TG, leptin, adiponectin, and resistin will be analyzed as dependent variables. Baseline variables that differ between groups will be included as covariates in the analysis. Dietary intake of macronutrients as estimated from 4-day food records, estimated energy expenditures, eating behaviors, health perceptions, and ratings of SCT mediators also will be compared among groups. A 3 x 5 (3 treatment groups x 5 time intervals) ANOVA with repeated measures on the time factor will be used to assess differences in outcomes within and between treatment groups over time. The group by time interaction will be examined for differences in time trend among intervention groups. Tukey pairwise comparisons will be used in conjunction with ANOVA to detect differences between treatment groups.

Some attrition is expected, as participants may be unable to comply with the intervention or may choose not to continue participation in the study. Participants who withdraw from the intervention will be asked to complete any remaining data collection sessions, and these data will be included in the statistical analyses (ie, intention-to-treat model). Data also will be analyzed using measurements only from those participants who complete all testing sessions. Statistical tests will be two-tailed with significance set at *P*<.01 to reduce the potential for Type I error. All statistical analyses will be conducted using Statistical Package for the Social Sciences (version 22.0, 2013, IBM Corp, Armonk, NY, USA).

## Results

Eighty-one women completed baseline testing. Baseline descriptive characteristics of the sample are displayed in Tables 1 and 2. Overall, these women were highly educated, with a majority of participants having at least a 4-year college degree. The racial/ethnic breakdown was reflective of the larger population, with non-Hispanic whites representing the majority. Age range was 18-45 years, and BMI range was 18.5-49.6 kg/m^2^. On average, participants were overweight and normotensive. Participants have been recruited, enrolled, and randomized to one of the three intervention groups. Education sessions will continue through August 2015, and results are expected by early 2016.

After 8 weeks, the halfway point for weekly education sessions, 75 (93%) of the original sample remained in the study. For those randomized to the two intervention groups, 49 (89%) women were still enrolled.

**Table 1:** Baseline characteristics of women (n=81) participating in a 12-month weight gain prevention intervention and completing baseline testing.

Characteristic	All participants mean (SD)
Age (years)	31.4 (8.1)
Height (cm)	165.2 (5.9)
Weight (kg)	76.1 (19.0)
Body mass index (kg/m^2^)	27.9 (6.8)
Waist circumference (cm)	83.3 (13.6)
Hip circumference (cm)	110.7 (14.6)
Waist: hip ratio	0.8 (0.1)
Body fat (%)	34.6 (9.1)
Fat mass (kg)	27.9 (14.2)
Fat free mass (kg)	48.2 (5.4)
Systolic blood pressure (mmHg)	106.2 (11.0)
Diastolic blood pressure (mmHg)	70.9 (9.9)
Resting heart rate (bpm)	65.8 (6.0)

**Table 2:** Demographic and education characteristics of all women (n=81) randomized to all groups.

Characteristic		All participants No (%)
**Education**		
	High school graduate	2 (3)
	Some college	15 (18)
	2-year associate degree/graduate	2 (3)
	4-year college degree/graduate	21 (26)
	Some graduate school	8 (10)
	Master’s degree	27 (33)
	Doctorate degree	6 (7)
**Race/ethnicity**		
	White, non-Hispanic	53 (66)
	Black, non-Hispanic	10 (12)
	Asian	8 (10)
	Non-white Hispanic or Latino	4 (5)
	Other (including multiracial)	6 (7)
**Total annual household income**		
	<$15,000	7 (9)
	$15,000 - $49,999	30 (37)
	$50,000 – $99,999	25 (31)
	>$100,000 – $199,999	18 (22)
	No response	1 (1)

## Discussion

### Principal Findings

The importance of weight gain prevention and maintenance of current weight has recently been recognized by the American College of Sports Medicine [18] and Healthy People 2020 [46] as critical; yet, there are currently no treatment guidelines for weight gain prevention. The gap in the understanding of the determinants, facilitators, and barriers to weight gain prevention likely exists due to the limited number of studies addressing prospective weight changes in adulthood. Awareness and identification of the determinants of weight gain prevention are necessary in order to increase the practicality of weight gain prevention for managing obesity.

While it may seem counterintuitive to promote weight gain prevention in overweight and obese individuals rather than weight loss, weight gain prevention is relevant for individuals of all BMI categories [47]. Preventing weight gain over time offers the opportunity to slow the progression of overweight and obesity and to avoid further exacerbations related to excess body weight in individuals who are already overweight or obese [48]. Additionally, weight gain prevention may require less intensive treatment than that required to achieve weight loss [2], and may be more successful in the long term as it avoids the problems associated with weight loss and its maintenance [27]. Weight maintenance, regardless of whether an individual is normal weight, overweight or obese, may be more beneficial and practical than repeated, minimally successful weight-loss attempts. While modest weight losses of 5-10% of body weight have significant effects on risk factors of disease, these benefits may be ameliorated with weight regain. Even with weight loss, metabolically healthy obese individuals may not show improvement in health outcomes, and weight loss in these individuals may promote weight cycling, or periods of weight loss followed by weight gain, which may have detrimental effects on mental, metabolic, and psychological outcomes [48-52]. Further, the adverse effects associated with weight cycling may be as harmful as maintenance of a high “unhealthy” body weight [51]. However, a recent study by Mason and colleagues [53] found that weight cycling was not associated with negative metabolic outcomes and a history of weight cycling was not related to the ability to lose and successfully maintain weight in the long term.

In a recent qualitative study of women who completed a weight-loss intervention conducted by a registered dietitian, women perceived the registered dietitian to be a credible source of nutrition information and found lack of access to a registered dietitian following completion of the intervention to be a barrier to weight-loss maintenance [29]. As a credible source of nutrition information [37], registered dietitians have a specialized skill set to support and encourage sustainable behavior changes to achieve weight management. Registered dietitians are generally regarded as the experts in weight management, but no studies have compared registered dietitians to other health professionals in the delivery of weight management information. The current study will fill this scientific gap by testing the ability of the registered dietitian to promote weight gain prevention as compared to an untrained professional. If registered dietitians are more effective in promoting weight gain prevention, these findings will support the notion that registered dietitians should be at the forefront in helping individuals attain successful weight management.

Women in the qualitative study [29] identified social support, basic nutrition education, accountability to others, self-motivation, mindfulness and awareness of food choices, planning ahead, portion control, and exercise as facilitators to weight loss and weight-loss maintenance while health status changes, environmental pressures, life transitions, absence of social support, lack of accountability, and internal factors were perceived as barriers. Additionally, women expressed their desire for continual contact with the registered dietitian as well as the group support offered by the education sessions [29].

Although there is no standardized definition of weight gain prevention or weight maintenance, a weight change of ±3% from baseline weight will be considered successful weight gain prevention. A 3% change criterion allows for normal day-to-day fluctuations that may result from measurement error, clothing, food consumption, and/or fluid balance [54].

### Limitations

Results from this study will be limited in generalizability to premenopausal women. Future research should examine pre- and post-menopausal women, as these physiological changes appear to be other critical life stage intervals for weight gain. Weight gain prevention should also be examined exclusively in men, as determinants of and strategies for weight gain prevention may differ between men and women. Further, our results may be limited by the length of the study, as the current intervention is only for one year. Surveys, testing sessions or focus groups following completion of the intervention may be useful in order to garner more information about the feasibility of long-term weight gain prevention. There are limitations with using self-reported dietary intake and physical activity; however, participants have been taught to accurately complete food records, and accelerometry data will be used to validate written physical activity records. Finally, our intervention contains multiple components that address weight gain prevention, and the study design does not allow for examination of independent effects of the different elements of this weight gain prevention intervention. Investigator-designed surveys will be used to assist with determining the effects of individual intervention components.

### Conclusions

The current study targets women who are at greater risk for weight gain compared to men; with the goal to help further the understanding of the determinants of weight gain prevention [29]. This study will fill a scientific gap in testing the ability of a registered dietitian to promote weight gain prevention as compared to another health professional that lacks formal nutrition and dietetics training. Although several studies have explored weight gain prevention with limited success, this may be the first study that targets young adult and midlife women of all weight status categories (normal weight, overweight, obese) and focuses on prospective weight gain prevention. Results of this research will be expected to have implications for policy development and recommendations for decreasing the burden of overweight and obesity in the United States through weight gain prevention.

## Multimedia Appendix 1

CONSORT-EHEALTH checklist V1.6.2 [55].

## Abbreviations

BF%: body fat percentage
BMI: body mass index
CON: control group
CSG: counselor group
CV: coefficients of variations
FM: fat mass
GOAL: Groningen Overweight and Lifestyle
Grit-S: Short Grit Scale
HDL-C: high-density lipoprotein cholesterol
IRB: Institutional Review Board
LDL-C: low-density lipoprotein cholesterol
MET: metabolic equivalent
NDSR: Nutrition Data System for Research
POP: Pound of Prevention
RDG: registered dietitian group
SCT: social cognitive theory
SF-36: Short-Form 36 Health Survey
SNAP: Study of Novel Approaches to Weight Gain Prevention
TG: triacylglycerides
UIUC: University of Illinois at Urbana-Champaign
