# Supplementary material for: Determinants of Weight Gain Prevention in Young Adult and Midlife Women: Study Design and Protocol of a Randomized Controlled Trial
Source: JMIR Res Protoc. 2015 Mar 26;4(1):e36. doi: 10.2196/resprot.4008 (PMC4392173; doi:10.2196/resprot.4008)
Supplement: Supplementary file 1 [file resprot_v4i1e36_app1.pdf]

CONSORT-EHEALTH Checklist V1.6.2 Report

(based on CONSORT-EHEALTH V1.6), available at [http://tinyurl.com/consort-ehealth-v1-6].

4008

Date completed

11/3/2014 21:23:59

by

Sharon M. Nickols-Richardson

"Determinants of weight gain prevention in young adult and midlife women: study design and protocol of a randomized controlled trial"

TITLE

1a-i) Identify the mode of delivery in the title

The mode of delivery is an in-person educational session; therefore, this item is not applicable or relevant to the current study.

1a-ii) Non-web-based components or important co-interventions in title

The mode of delivery is an in-person educational session; therefore, this item is not applicable or relevant to the current study.

1a-iii) Primary condition or target group in the title

"Determinants of weight gain prevention in young adult and midlife women: study design and protocol of a randomized controlled trial"

ABSTRACT

1b-i) Key features/functionalities/components of the intervention and comparator in the METHODS section of the ABSTRACT

"The objective of this randomized controlled trial (RCT) is to compare a weight gain prevention intervention delivered by the registered dietitian versus counselor."

"This is a 12-month parallel-arm weight gain prevention RCT designed to increase self-efficacy, self-regulation, outcome expectations and family and social support through the use of a nutrition education intervention in women, ages 18-45 years from the Urbana-Champaign (Illinois, USA) area. Women have been randomized to registered dietitian, counselor or wait-list control groups (August 2014) and are undergoing weekly nutrition education sessions for four months, followed by monthly sessions for eight months (through August 2015)."

1b-ii) Level of human involvement in the METHODS section of the ABSTRACT

"The objective of this randomized controlled trial (RCT) is to compare a weight gain prevention intervention delivered by the registered dietitian versus counselor."

"This is a 12-month parallel-arm weight gain prevention RCT designed to increase self-efficacy, self-regulation, outcome expectations and family and social support through the use of a nutrition education intervention in women, ages 18-45 years from the Urbana-Champaign (Illinois, USA) area. Women have been randomized to registered dietitian, counselor or wait-list control groups (August 2014) and are undergoing weekly nutrition education sessions for four months, followed by monthly sessions for eight months (through August 2015)."

1b-iii) Open vs. closed, web-based (self-assessment) vs. face-to-face assessments in the METHODS section of the ABSTRACT

"This is a 12-month parallel-arm weight gain prevention RCT designed to increase self-efficacy, self-regulation, outcome expectations and family and social support through the use of a nutrition education intervention in women, ages 18-45 years from the Urbana-Champaign (Illinois, USA) area. Women have been randomized to registered dietitian, counselor or wait-list control groups (August 2014) and are undergoing weekly nutrition education sessions for four months, followed by monthly sessions for eight months (through August 2015). Outcome measures, including: (1) dietary intake; (2) physical activity; (3) anthropometric and blood pressure measurements; (4) biochemical markers of health; (5) eating behaviors and health perceptions, and (6) mediators of behavior change, were collected before the intervention began (baseline) and will be collected at 3, 6, 9 and 12 months of the study."

1b-iv) RESULTS section in abstract must contain use data

"Eighty-seven women have been randomized to intervention groups, and 81 women have completed week 1 of the study. Results are expected in early 2016."

1b-v) CONCLUSIONS/DISCUSSION in abstract for negative trials

This is not relevant or applicable yet, as the study is currently in progress.

INTRODUCTION

2a-i) Problem and the type of system/solution

"Small weight gains over time, approximating 1-2 pounds per year [1,2], contribute to the development of overweight and obesity. Once established, obesity is difficult to treat [3], as reduction of excess body weight is rarely effective in the long term. Short-term weight loss can be achieved by a variety of methods, but few of these approaches are sustainable and effective in facilitating permanent weight loss [4-10]. On average, individuals adhere to weight-loss programs for approximately six months [11]; following weight loss, most individuals regain half of the weight lost within one year, and return to baseline weight within 3-5 years [11-13]. Weight gain prevention, on the other hand, avoids the difficulties that may accompany weight loss and weight-loss maintenance and offers an alternative option for weight management."

This is a stand-alone intervention of weight gain prevention, targeted for women.

2a-ii) Scientific background, rationale: What is known about the (type of) system

"To reduce disease risk and improve overall health, effective weight gain prevention is essential; however, few interventions have successfully examined weight gain prevention and little is known about the determinants of and strategies for preventing weight gain over the long term. Much of the existing research has focused on treatment of overweight and obesity through reduction of excess body weight [14,15] or prevention of weight regain following weight loss [16-20]."

"Without complete knowledge of the determinants of and strategies for weight gain prevention, public health will remain at risk for complications and costs related to overweight and obesity. Weight gain prevention offers a primary strategy for weight management and obesity prevention [24,26]."

METHODS

3a) CONSORT: Description of trial design (such as parallel, factorial) including allocation ratio

"The current study aims to identify determinants of weight gain prevention in young adult and midlife premenopausal women through a 1-year weight gain prevention intervention that includes nutrition education. We hypothesize that compared to a wait-list control group, women who participate in a weight gain prevention intervention designed to increase self-efficacy, self-regulation, outcome expectations and family and social support will maintain current body weight during a 12-month period. It is further hypothesized that women in an intervention group led by registered dietitians will have lesser weight gain across 12 months compared to women in an intervention group led by counselors."

3b) CONSORT: Important changes to methods after trial commencement (such as eligibility criteria), with reasons

This item is not applicable or relevant for the current study, because no changes have been made to the methods after trial commencement.

3b-i) Bug fixes, Downtimes, Content Changes

This item is not applicable or relevant to the current study, because there have been no "bug fixes, downtimes, or content changes."

4a) CONSORT: Eligibility criteria for participants

"The current study included premenopausal women between the ages of 18-45 years with a BMI of >18.5 kg/m2. There were no additional criteria for body weight and BMI to ensure participation by women from a range of weight status categories. Further inclusion criteria included eumenorrhea (≥8 menstrual cycles/year), score of <50 on the Zung Self-Rating Depression Scale/Status Inventory [29] and no self-reported metabolic, cardiovascular or musculoskeletal diseases or use of medications or supplements to manage a chronic health condition. Exclusion criteria included women who currently smoked, were pregnant or attempting to become pregnant or were currently lactating. Women using medications influencing weight regulation, such as steroid or thyroid hormones or oral contraceptives, were excluded if use was for <2 months before the start of the study. Gastric bypass surgery was also an exclusion criterion."

4a-i) Computer / Internet literacy

This is not applicable or relevant to the current study.

4a-ii) Open vs. closed, web-based vs. face-to-face assessments:

"Participants were recruited by word-of-mouth, electronic-mail messages and posted flyers from the University of Illinois campus and the Urbana-Champaign (IL, USA) communities."

"During the 1-year study, women randomized to the RDG and CSG groups will attend a total of 24, 1-hour nutrition education sessions that are based on effective weight-loss programs/plans and address energy balance through sustainable diet, exercise and behavior modifications [30-36]. These sessions will be held weekly for 16 weeks (months 1-4) and monthly thereafter (months 5-12) [37]. Vegetable consumption, planning ahead for food intake and portion control will be emphasized [30,31], and general nutrition information, eating away from home, food selection, food preparation and recipe modification also will be addressed [30-33]. Other topics will include fitness and physical activity, culinary skills, breakfast consumption, healthy snacking and beverage choices, nutrient density, family menu planning and grocery shopping. Problem solving, motivational concerns and stress management will be encouraged [30-36]. Education sessions will relay constructs of the Social Cognitive Theory (SCT) [37]."

"Education sessions will follow a three-part format. Each session will begin with a brief review of information covered in the previous session and will address participant progress, including successes, challenges and questions. Next, the leader will deliver the nutrition education component of the session using an interactive group discussion format. Participants will be provided with handouts addressing food choices, dietary patterns, menu plans and other information pertaining to the lesson. Finally, the content for the lesson will be summarized and participants will have a chance to ask questions, address concerns and set specific behavioral goals for the next session. Education sessions will be randomly selected for evaluation by a process observer who will rate the sessions based on investigator-established criteria."

"Four registered dietitians will deliver the intervention to women in the RDG group. All women in the RDG group will equitably interact with all four registered dietitians across the study. Four counselors will deliver the intervention to women in the CSG group, with these women having equitable interaction with all four counselors across the study. The credentials of the professional delivering the intervention will not be revealed to participants until after completion of the study. The registered dietitians are all female and have been practicing for <5 years; the counselors are all female and are graduate teaching assistants at UIUC in programs unrelated to nutrition or dietetics. Compliance will be defined as attendance of >85% of education sessions. If women are unable to attend an education session, virtual make-up sessions will be offered, along with a quiz. Completion and return of the quiz will indicate that the materials were studied and reviewed and that the participant was compliant."

"Before the intervention (baseline), data on dietary intake, physical activity, anthropometric and blood pressure measurements, biochemical markers of health, eating behaviors and health perceptions, and SCT mediators of behavioral change were collected. These outcome measures also will be obtained at 3, 6, 9 and 12 months."

4a-iii) Information giving during recruitment

"A total of 330 women responded to recruitment methods, between June and August 2014. Of these, 266 women met pre-screening criteria [appropriate age, body mass index (BMI), and desire to prevent weight gain] and received screening materials including a Medical History Form, Zung Self-Rating Depression Scale/Status Inventory [29] and Informed Consent. A total of 146 women returned screening materials, which were reviewed by investigators. One hundred two women met eligibility criteria for participation, and 87 women were randomized, with 81 women completing baseline testing."

Every participant was required to provide written informed consent. The Informed Consent Form was required to include:
*Name of institution;
*Title of project;
*Name of principal investigator;
*Purpose of the study;
*Procedures to be followed;
*Discomforts and risks;
*Abnormal test results;
*Benefits to individual;
*Benefits to society;
*Duration/time of the procedures and study;
*Statement of confidentiality;
*Right to ask questions;
*Payment for participation;
*Voluntary participation;
*Event of injury;
*Signature of participant and date; and
*Signature of investigator and date.

4b) CONSORT: Settings and locations where the data were collected

"Participants were recruited by word-of-mouth, electronic-mail messages and posted flyers from the University of Illinois campus and the Urbana-Champaign (IL, USA) communities."

"The Institutional Review Board (IRB) for the Protection of Human Subjects at the University of Illinois at Urbana-Champaign (UIUC) approved the study protocol (UIUC IRB#14397)."

4b-i) Report if outcomes were (self-)assessed through online questionnaires

"Outcome Measures
Before the intervention (baseline), data on dietary intake, physical activity, anthropometric and blood pressure measurements, biochemical markers of health, eating behaviors and health perceptions, and SCT mediators of behavioral change were collected. These outcome measures also will be obtained at 3, 6, 9 and 12 months."

"Dietary Intake and Physical Activity Assessment
Participants were taught to accurately complete 4-day food records and the Stanford 7-day Physical Activity Recall Scale [38]. To ensure accuracy in recording foods and beverage consumption, handouts containing examples of standard serving sizes were provided. Participants recorded all foods and beverages consumed for three non-consecutive weekdays and one weekend day before the baseline testing session [31]. Four-day food records will be analyzed using the Nutrition Data System for Research (NDSR) dietary analysis software (Nutrition Coordinating Center, Minneapolis, MN, USA) to estimate average daily dietary intake of total energy (kcal/d), carbohydrate (g/d), protein (g/d), fat (g/d) and fiber (g/d) in addition to intake by food groups (svgs/d)."

"For seven consecutive days before the baseline testing session, participants recorded the number of hours slept, spent in front of a television or computer screen and engaged in moderate, hard and very hard physical activity [31]. Participants wore accelerometers at the waist, wrist or ankle during all waking hours for seven consecutive days while also recording physical activity to provide an objective assessment of energy expenditure. Approximately 70% of participants in each group wore accelerometers as accelerometers were not available for all individuals. Physical activity records will be analyzed by summing total hours of moderate, hard and very hard activity and dividing by seven to estimate hours of physical activity per day. These records will be further analyzed by converting activities into METs (hr/d), which will be evaluated as light activity (1-3 METs), moderate activity (>3-6 METs), and vigorous activity (>6 METs) to estimate the number of calories expended per day. Accelerometry data will be analyzed using ActiLife 6.11 (ActiGraph, Pensacola, FL, USA) to estimate the number of calories expended per day, the MET rate per day, and the length of time (minutes) spent in sedentary, light, moderate, vigorous and very vigorous activities."

"Anthropometric and Blood Pressure Measurements
Baseline standing height (cm) was recorded to the nearest 0.1 cm using a calibrated scale-mounted stadiometer (Seca 700, Hanover, MD, USA). Body weight (kg) was measured using a calibrated balance-beam scale (Seca 700) to the nearest 0.1 kg. BMI (kg/m2) was calculated using height and body weight measurements. A retractable measuring tape (Gulik II, Country Technology, Inc., Gay Mills, WI) was used to measure waist (cm) and hip (cm) circumferences, in duplicate, to the nearest 0.1 cm according to standard protocol [30]. Waist circumference was measured at the narrowest point of the waist, approximately one inch above the navel, and hip circumference was measured at the widest part of the buttocks [31]. Waist and hip circumference measurements were averaged to obtain a single value for each site; these values were used to calculate the waist:hip ratio. Fat mass (kg) and body fat percentage (BF%) were measured using a Tanita scale (410GS, Arlington Heights, IL, USA)."

"Seated systolic and diastolic blood pressure (mm Hg) were measured by a trained study investigator using a standard sphygmomanometer (Baumanometer® Desk Model, Copiague, NY, USA) following a 5-minute rest period. Blood pressure measurements were taken in duplicate with a 2-3-minute rest period between readings; mean systolic arterial pressure values and diastolic arterial pressure values will be used in data analyses. Resting heart rate was also measured after the 5-minute rest period."

"Biochemical Markers of Health
Venous blood samples (~30 mL) were collected by a trained phlebotomist between 0700-0930 hour after a 12-hour fast. Whole blood samples were processed and stored at -80ºC. Serum will be analyzed for concentrations of insulin, glucose, glycosylated hemoglobin, total cholesterol, high-density lipoprotein-cholesterol (HDL-C), low-density lipoprotein-cholesterol (LDL-C), triacylglycerides (TG), leptin, adiponectin and resisting."

"Serum insulin (µU/mL) (LINCO Research, St. Charles, MO, USA) will be measured using enzyme-linked immunosorbent assay (ELISA), and serum glucose (mg/dL) (Stanbio Labs, Boerne, TX, USA) will be measured by spectrophotometry. Direct glycosylated hemoglobin (HbA1C, %) will be measured by immunoassay (Stanbio Labs). Total cholesterol (mg/dL), HDL-C (mg/dL) and TG (mg/dL) concentrations will be measured by spectrophotometry using the total cholesterol, HDL-C and TG kits, respectively (Stanbio Labs). Total cholesterol, HDL-C and TG concentrations will be used to calculate LDL-C concentration (mg/dL) using the equation: LDL-C = total cholesterol – HDL-C – (TG/5) [39]. Serum leptin (ng/mL), adiponectin (ng/mL) and resistin (ng/mL) will be measured using ELISA (R&D Systems, Minneapolis, MN, USA). All serum samples for each biomarker will be analyzed in duplicate at corresponding study intervals. Intra- and inter-assay coefficients of variations (CV) are <15% for all kits."

"Eating Behaviors, Health Perceptions and SCT Mediators of Behavioral Change
Participants completed questionnaires designed to evaluate eating behaviors, health perceptions, perseverance and SCT mediators. The Eating Inventory [40] will evaluate ratings of cognitive eating restraint, hunger and disinhibition. The Short-Form 36 Health Survey (SF-36) [41] will assess self-reported health issues. Perseverance will be examined using the Short Grit Scale (Grit-S) [42], and an investigator-designed questionnaire will evaluate SCT mediators, including self-efficacy, outcome expectations, self-regulation and social and family support. Standard scoring and interpretation methods will be used to evaluate all questionnaires [40-42]."

4b-ii) Report how institutional affiliations are displayed

This is not applicable or relevant for the current study, as educational sessions and data collection sessions were held on the campus of the institution.

5) CONSORT: Describe the interventions for each group with sufficient details to allow replication, including how and when they were actually administered

5-i) Mention names, credential, affiliations of the developers, sponsors, and owners

This does not apply to the current study. Software was not developed for this intervention.

5-ii) Describe the history/development process

This does not apply to the current study, as software was not developed.

5-iii) Revisions and updating

This does not apply to the current study, as software was not developed. The educational sessions were tested and used in a previous study.

"31. Nickols-Richardson SM, Piehowski KE, Metzgar CJ, Miller DL, Preston AG. Changes in body weight, blood pressure and selected metabolic biomarkers with an energy-restricted diet including twice daily sweet snacks and once daily sugar-free beverage. Nutr Res Pract 2014 Dec;8(6):in press."

5-iv) Quality assurance methods

"Education sessions will be randomly selected for evaluation by a process observer who will rate the sessions based on investigator-established criteria."

"Compliance will be defined as attendance of >85% of education sessions."

"Intra- and inter-assay coefficients of variations (CV) are <15% for all kits."

5-v) Ensure replicability by publishing the source code, and/or providing screenshots/screen-capture video, and/or providing flowcharts of the algorithms used

"Participants were taught to accurately complete 4-day food records and the Stanford 7-day Physical Activity Recall Scale [38]. To ensure accuracy in recording foods and beverage consumption, handouts containing examples of standard serving sizes were provided. Participants recorded all foods and beverages consumed for three non-consecutive weekdays and one weekend day before the baseline testing session [31]. Four-day food records will be analyzed using the Nutrition Data System for Research (NDSR) dietary analysis software (Nutrition Coordinating Center, Minneapolis, MN, USA) to estimate average daily dietary intake of total energy (kcal/d), carbohydrate (g/d), protein (g/d), fat (g/d) and fiber (g/d) in addition to intake by food groups (svgs/d)."

"Participants wore accelerometers at the waist, wrist or ankle during all waking hours for seven consecutive days while also recording physical activity to provide an objective assessment of energy expenditure."

"Serum insulin (µU/mL) (LINCO Research, St. Charles, MO, USA) will be measured using enzyme-linked immunosorbent assay (ELISA), and serum glucose (mg/dL) (Stanbio Labs, Boerne, TX, USA) will be measured by spectrophotometry. Direct glycosylated hemoglobin (HbA1C, %) will be measured by immunoassay (Stanbio Labs). Total cholesterol (mg/dL), HDL-C (mg/dL) and TG (mg/dL) concentrations will be measured by spectrophotometry using the total cholesterol, HDL-C and TG kits, respectively (Stanbio Labs). Total cholesterol, HDL-C and TG concentrations will be used to calculate LDL-C concentration (mg/dL) using the equation: LDL-C = total cholesterol – HDL-C – (TG/5) [39]. Serum leptin (ng/mL), adiponectin (ng/mL) and resistin (ng/mL) will be measured using ELISA (R&D Systems, Minneapolis, MN, USA). All serum samples for each biomarker will be analyzed in duplicate at corresponding study intervals. Intra- and inter-assay coefficients of variations (CV) are <15% for all kits."

"The Eating Inventory [40] will evaluate ratings of cognitive eating restraint, hunger and disinhibition. The Short-Form 36 Health Survey (SF-36) [41] will assess self-reported health issues. Perseverance will be examined using the Short Grit Scale (Grit-S) [42], and an investigator-designed questionnaire will evaluate SCT mediators, including self-efficacy, outcome expectations, self-regulation and social and family support. Standard scoring and interpretation methods will be used to evaluate all questionnaires [40-42]."

5-vi) Digital preservation

This does not apply to the current study.

5-vii) Access

"Education sessions will follow a three-part format. Each session will begin with a brief review of information covered in the previous session and will address participant progress, including successes, challenges and questions. Next, the leader will deliver the nutrition education component of the session using an interactive group discussion format. Participants will be provided with handouts addressing food choices, dietary patterns, menu plans and other information pertaining to the lesson. Finally, the content for the lesson will be summarized and participants will have a chance to ask questions, address concerns and set specific behavioral goals for the next session. Education sessions will be randomly selected for evaluation by a process observer who will rate the sessions based on investigator-established criteria.
Four registered dietitians will deliver the intervention to women in the RDG group. All women in the RDG group will equitably interact with all four registered dietitians across the study. Four counselors will deliver the intervention to women in the CSG group, with these women having equitable interaction with all four counselors across the study. The credentials of the professional delivering the intervention will not be revealed to participants until after completion of the study. The registered dietitians are all female and have been practicing for <5 years; the counselors are all female and are graduate teaching assistants at UIUC in programs unrelated to nutrition or dietetics. Compliance will be defined as attendance of >85% of education sessions. If women are unable to attend an education session, virtual make-up sessions will be offered, along with a quiz. Completion and return of the quiz will indicate that the materials were studied and reviewed and that the participant was compliant."

5-viii) Mode of delivery, features/functionalities/components of the intervention and comparator, and the theoretical framework

"During the 1-year study, women randomized to the RDG and CSG groups will attend a total of 24, 1-hour nutrition education sessions that are based on effective weight-loss programs/plans and address energy balance through sustainable diet, exercise and behavior modifications [30-36]. These sessions will be held weekly for 16 weeks (months 1-4) and monthly thereafter (months 5-12) [37]. Vegetable consumption, planning ahead for food intake and portion control will be emphasized [30,31], and general nutrition information, eating away from home, food selection, food preparation and recipe modification also will be addressed [30-33]. Other topics will include fitness and physical activity, culinary skills, breakfast consumption, healthy snacking and beverage choices, nutrient density, family menu planning and grocery shopping. Problem solving, motivational concerns and stress management will be encouraged [30-36]. Education sessions will relay constructs of the Social Cognitive Theory (SCT) [37].
Education sessions will follow a three-part format. Each session will begin with a brief review of information covered in the previous session and will address participant progress, including successes, challenges and questions. Next, the leader will deliver the nutrition education component of the session using an interactive group discussion format. Participants will be provided with handouts addressing food choices, dietary patterns, menu plans and other information pertaining to the lesson. Finally, the content for the lesson will be summarized and participants will have a chance to ask questions, address concerns and set specific behavioral goals for the next session. Education sessions will be randomly selected for evaluation by a process observer who will rate the sessions based on investigator-established criteria.
Four registered dietitians will deliver the intervention to women in the RDG group. All women in the RDG group will equitably interact with all four registered dietitians across the study. Four counselors will deliver the intervention to women in the CSG group, with these women having equitable interaction with all four counselors across the study. The credentials of the professional delivering the intervention will not be revealed to participants until after completion of the study. The registered dietitians are all female and have been practicing for <5 years; the counselors are all female and are graduate teaching assistants at UIUC in programs unrelated to nutrition or dietetics. Compliance will be defined as attendance of >85% of education sessions. If women are unable to attend an education session, virtual make-up sessions will be offered, along with a quiz. Completion and return of the quiz will indicate that the materials were studied and reviewed and that the participant was compliant."

5-ix) Describe use parameters

Educational sessions were held once per week for 4 months and then once per month for 8 months, for a total of 24, 1-hour sessions.

5-x) Clarify the level of human involvement

Human involvement included 1, 1-hour education session per week for 4 months, followed by 1, 1-hour education session per month for 8 months.

5-xi) Report any prompts/reminders used

When women indicated that they were unable to attend an education session, an investigator provided materials from the education session.

5-xii) Describe any co-interventions (incl. training/support)

This does not apply to the current study.

6a) CONSORT: Completely defined pre-specified primary and secondary outcome measures, including how and when they were assessed

"Before the intervention (baseline), data on dietary intake, physical activity, anthropometric and blood pressure measurements, biochemical markers of health, eating behaviors and health perceptions, and SCT mediators of behavioral change were collected. These outcome measures also will be obtained at 3, 6, 9 and 12 months."

6a-i) Online questionnaires: describe if they were validated for online use and apply CHERRIES items to describe how the questionnaires were designed/deployed

Online questionnaires were not used in the current study.

6a-ii) Describe whether and how "use" (including intensity of use/dosage) was defined/measured/monitored

Based on our previous work, weekly educational sessions for 4 months, followed by monthly educational sessions were determined.

6a-iii) Describe whether, how, and when qualitative feedback from participants was obtained

Qualitative feedback from participants has not been collected.

6b) CONSORT: Any changes to trial outcomes after the trial commenced, with reasons

This does not apply to the current study. There have been no changes to the trial outcomes after commencement of the study.

7a) CONSORT: How sample size was determined

7a-i) Describe whether and how expected attrition was taken into account when calculating the sample size

Attrition was expected and accounted for, such that the sample was over-recruited to allow for attrition.

7b) CONSORT: When applicable, explanation of any interim analyses and stopping guidelines

This does not apply to the current study, as educational sessions are not expected to produce results that would lead to stopping the study.

8a) CONSORT: Method used to generate the random allocation sequence

Participants were randomized to intervention group, using a random number generator.

8b) CONSORT: Type of randomisation; details of any restriction (such as blocking and block size)

This does not apply as special restrictions were not used.

9) CONSORT: Mechanism used to implement the random allocation sequence (such as sequentially numbered containers), describing any steps taken to conceal the sequence until interventions were assigned

Randomization was conducted as described previously.

10) CONSORT: Who generated the random allocation sequence, who enrolled participants, and who assigned participants to interventions

Investigators used an electronic random allocation sequencer; investigators enrolled participants, and used a random number generator to assign participants to interventions.

11a) CONSORT: Blinding - If done, who was blinded after assignment to interventions (for example, participants, care providers, those assessing outcomes) and how

11a-i) Specify who was blinded, and who wasn't

Participants were blinded to intervention group, with the exception of the wait-list control group. Participants who are receiving the educational intervention have not been told whether the are in the registered dietitian or counselor group. Wait-list control group participants are aware that they are not receiving the educational intervention.

11a-ii) Discuss e.g., whether participants knew which intervention was the "intervention of interest" and which one was the "comparator"

"The credentials of the professional delivering the intervention will not be revealed to participants until after completion of the study."

11b) CONSORT: If relevant, description of the similarity of interventions

"During the 1-year study, women randomized to the RDG and CSG groups will attend a total of 24, 1-hour nutrition education sessions that are based on effective weight-loss programs/plans and address energy balance through sustainable diet, exercise and behavior modifications [30-36]. These sessions will be held weekly for 16 weeks (months 1-4) and monthly thereafter (months 5-12) [37]. Vegetable consumption, planning ahead for food intake and portion control will be emphasized [30,31], and general nutrition information, eating away from home, food selection, food preparation and recipe modification also will be addressed [30-33]. Other topics will include fitness and physical activity, culinary skills, breakfast consumption, healthy snacking and beverage choices, nutrient density, family menu planning and grocery shopping. Problem solving, motivational concerns and stress management will be encouraged [30-36]. Education sessions will relay constructs of the Social Cognitive Theory (SCT) [37]."

"Education sessions will follow a three-part format. Each session will begin with a brief review of information covered in the previous session and will address participant progress, including successes, challenges and questions. Next, the leader will deliver the nutrition education component of the session using an interactive group discussion format. Participants will be provided with handouts addressing food choices, dietary patterns, menu plans and other information pertaining to the lesson. Finally, the content for the lesson will be summarized and participants will have a chance to ask questions, address concerns and set specific behavioral goals for the next session. Education sessions will be randomly selected for evaluation by a process observer who will rate the sessions based on investigator-established criteria."

"Four registered dietitians will deliver the intervention to women in the RDG group. All women in the RDG group will equitably interact with all four registered dietitians across the study. Four counselors will deliver the intervention to women in the CSG group, with these women having equitable interaction with all four counselors across the study. The credentials of the professional delivering the intervention will not be revealed to participants until after completion of the study. The registered dietitians are all female and have been practicing for <5 years; the counselors are all female and are graduate teaching assistants at UIUC in programs unrelated to nutrition or dietetics. Compliance will be defined as attendance of >85% of education sessions. If women are unable to attend an education session, virtual make-up sessions will be offered, along with a quiz. Completion and return of the quiz will indicate that the materials were studied and reviewed and that the participant was compliant."

12a) CONSORT: Statistical methods used to compare groups for primary and secondary outcomes

"Baseline characteristics of study participants were characterized using descriptive statistics (means ± SD). Participants in the three intervention groups (Treatment) will participate in five data collection sessions at specified intervals (Time). The Shapiro-Wilk test for normality will be used to test for normality and homogeneity of variance within groups; data will be transformed if necessary. Body weight, BMI, waist:hip ratio, FM, BF%, systolic and diastolic blood pressure, serum insulin, glucose, HbA1C, TC, HDL-C, LDL-C, TG, leptin, adiponectin and resistin will be analyzed as dependent variables; baseline variables that differ between groups will be included as covariates in the analysis. Dietary intake of macronutrients as estimated from 4-day food records, estimated energy expenditures, eating behaviors, health perceptions and ratings of SCT mediators also will be compared among groups. A 3 x 5 (3 treatment groups x 5 time intervals) ANOVA with repeated measures on the time factor will be used to assess differences in outcomes within and between treatment groups over time. The group by time interaction will be examined for differences in time trend among intervention groups. Tukey pairwise comparisons will be used in conjunction with ANOVA to detect differences between treatment groups."

12a-i) Imputation techniques to deal with attrition / missing values

"Some attrition is expected, as participants may be unable to comply with the intervention or may choose not to continue participation in the study. Participants who withdraw from the intervention will be asked to complete any remaining data collection sessions, and these data will be included in the statistical analyses (i.e., intention-to-treat model). Data also will be analyzed using measurements only from those participants who complete all testing sessions."

12b) CONSORT: Methods for additional analyses, such as subgroup analyses and adjusted analyses

This is not applicable for the current study, as the statistical strategy has been described previously.

RESULTS

13a) CONSORT: For each group, the numbers of participants who were randomly assigned, received intended treatment, and were analysed for the primary outcome

"Eighty-one women completed baseline testing. Baseline descriptive characteristics of the sample are displayed in Tables 1 and 2. Overall, these women were fairly highly educated, with a majority of participants having at least a 4-year college degree. The racial/ethnic breakdown was reflective of the larger population, with non-Hispanic whites representing the majority. Age ranged from 18-45 years, and BMI ranged from 18.5-49.6 kg/m2. On average, participants were overweight and normotensive. Participants have been recruited, enrolled and randomized to one of the three intervention groups. Education sessions will continue through August 2015, and results are expected by early 2016."

13b) CONSORT: For each group, losses and exclusions after randomisation, together with reasons

"After 8 weeks, the halfway point for weekly education sessions, 92.6% of the original sample remained in the study. For women randomized to the two intervention groups, 89.1% were still enrolled."

A flow diagram has been included in the manuscript as Figure 1. This diagram includes explanations for attrition.

13b-i) Attrition diagram

Figure 1 addresses attrition.

14a) CONSORT: Dates defining the periods of recruitment and follow-up

"A total of 330 women responded to recruitment methods, between June and August 2014."

"Education sessions will continue through August 2015, and results are expected by early 2016."

14a-i) Indicate if critical “secular events” fell into the study period

No secular events have affected the study to date.

14b) CONSORT: Why the trial ended or was stopped (early)

This is not applicable to the current study as the trial has not ended and has not been stopped early.

15) CONSORT: A table showing baseline demographic and clinical characteristics for each group

This information is included in Table 1 and Table 2 of the manuscript.

15-i) Report demographics associated with digital divide issues

Digital divide is not a factor in this study.

16a) CONSORT: For each group, number of participants (denominator) included in each analysis and whether the analysis was by original assigned groups

16-i) Report multiple “denominators” and provide definitions

Sample size has been included in Figure 1 (flow diagram) and Table 1 and Table 2.

16-ii) Primary analysis should be intent-to-treat

"Some attrition is expected, as participants may be unable to comply with the intervention or may choose not to continue participation in the study. Participants who withdraw from the intervention will be asked to complete any remaining data collection sessions, and these data will be included in the statistical analyses (i.e., intention-to-treat model). Data also will be analyzed using measurements only from those participants who complete all testing sessions."

17a) CONSORT: For each primary and secondary outcome, results for each group, and the estimated effect size and its precision (such as 95% confidence interval)

"Statistical tests will be two-tailed with significance set at P<.01 to reduce the potential for Type I error."

"Baseline characteristics of study participants were characterized using descriptive statistics (means ± SD). Participants in the three intervention groups (Treatment) will participate in five data collection sessions at specified intervals (Time). The Shapiro-Wilk test for normality will be used to test for normality and homogeneity of variance within groups; data will be transformed if necessary. Body weight, BMI, waist:hip ratio, FM, BF%, systolic and diastolic blood pressure, serum insulin, glucose, HbA1C, TC, HDL-C, LDL-C, TG, leptin, adiponectin and resistin will be analyzed as dependent variables; baseline variables that differ between groups will be included as covariates in the analysis. Dietary intake of macronutrients as estimated from 4-day food records, estimated energy expenditures, eating behaviors, health perceptions and ratings of SCT mediators also will be compared among groups. A 3 x 5 (3 treatment groups x 5 time intervals) ANOVA with repeated measures on the time factor will be used to assess differences in outcomes within and between treatment groups over time. The group by time interaction will be examined for differences in time trend among intervention groups. Tukey pairwise comparisons will be used in conjunction with ANOVA to detect differences between treatment groups."

17a-i) Presentation of process outcomes such as metrics of use and intensity of use

One educational session is equal to one hour.

17b) CONSORT: For binary outcomes, presentation of both absolute and relative effect sizes is recommended

This is not applicable to the current study as effect size is not the primary analytical approach of interest. Effect size may be calculated if sample size and variation between groups indicates a need for this approach.

18) CONSORT: Results of any other analyses performed, including subgroup analyses and adjusted analyses, distinguishing pre-specified from exploratory

This is not applicable as outcomes data have not been analyzed.

18-i) Subgroup analysis of comparing only users

"Some attrition is expected, as participants may be unable to comply with the intervention or may choose not to continue participation in the study. Participants who withdraw from the intervention will be asked to complete any remaining data collection sessions, and these data will be included in the statistical analyses (i.e., intention-to-treat model). Data also will be analyzed using measurements only from those participants who complete all testing sessions."

19) CONSORT: All important harms or unintended effects in each group

Risks were addressed in the IRB protocol. There were no risks greater than in everyday life from participation in this study.

19-i) Include privacy breaches, technical problems

There have been no privacy breaches or technical problems to date.

19-ii) Include qualitative feedback from participants or observations from staff/researchers

This does not apply to the current study as this research is not a qualitative study about the process of research.

DISCUSSION

20) CONSORT: Trial limitations, addressing sources of potential bias, imprecision, multiplicity of analyses

20-i) Typical limitations in ehealth trials

"Statistical tests will be two-tailed with significance set at P<.01 to reduce the potential for Type I error."

21) CONSORT: Generalisability (external validity, applicability) of the trial findings

21-i) Generalizability to other populations

"Results from this study will be limited in generalizability to premenopausal women."

"Further, our results may be limited by the length of the study, as the current intervention is only for one year."

"There are limitations with using self-reported dietary intake and physical activity; however, participants have been taught to accurately complete food records, and accelerometry data will be used to validate written physical activity records. Finally, our intervention contains multiple components that address weight gain prevention, and the study design does not allow for examination of independent effects of the different elements of this weight gain prevention intervention."

21-ii) Discuss if there were elements in the RCT that would be different in a routine application setting

This does not apply to the current study, as the elements in the RCT are available in routine application settings.

22) CONSORT: Interpretation consistent with results, balancing benefits and harms, and considering other relevant evidence

22-i) Restate study questions and summarize the answers suggested by the data, starting with primary outcomes and process outcomes (use)

This is not applicable at this time as results are not available. The discussion indicates anticipated findings and how these expectations fit within the context of previously conducted research.

22-ii) Highlight unanswered new questions, suggest future research

This does not apply, as it is premature to speculate on unanswered new questions and future research, based on the study to date (still in progress).

Other information

23) CONSORT: Registration number and name of trial registry

There is no registration number or name of trial registry for this study.

24) CONSORT: Where the full trial protocol can be accessed, if available

The full trial protocol can be accessed in this manuscript, once published.

25) CONSORT: Sources of funding and other support (such as supply of drugs), role of funders

"This research is supported by a grant from the United States Department of Agriculture, National Institute of Food and Agriculture, Illinois Agricultural Experiment Station, #ILLU-698-337. Graduate research assistantship support is provided by The Hershey Company, Hershey, PA, USA."

The funding agencies had no role in any part of this study, other than providing research funding for materials, supplies, and assistantship support.

X26-i) Comment on ethics committee approval

"The Institutional Review Board (IRB) for the Protection of Human Subjects at the University of Illinois at Urbana-Champaign (UIUC) approved the study protocol (UIUC IRB#14397). Each participant provided written informed consent before study participation."

x26-ii) Outline informed consent procedures

"The Institutional Review Board (IRB) for the Protection of Human Subjects at the University of Illinois at Urbana-Champaign (UIUC) approved the study protocol (UIUC IRB#14397). Each participant provided written informed consent before study participation."

The Informed Consent Form is required to include:
*Name of institution;
*Title of project;
*Name of principal investigator;
*Purpose of the study;
*Procedures to be followed;
*Discomforts and risks;
*Abnormal test results;
*Benefits to individual;
*Benefits to society;
*Duration/time of the procedures and study;
*Statement of confidentiality;
*Right to ask questions;
*Payment for participation;
*Voluntary participation;
*Event of injury;
*Signature of participant and date; and
*Signature of investigator and date.

X26-iii) Safety and security procedures

Safety and security was addressed in the IRB protocol that was approved by the institution at which this study is being conducted. The University of Illinois' IRB approved the study protocol (IRB#14397), which addressed privacy and confidentiality.

X27-i) State the relation of the study team towards the system being evaluated

"This research is supported by a grant from the United States Department of Agriculture, National Institute of Food and Agriculture, Illinois Agricultural Experiment Station, #ILLU-698-337. Graduate research assistantship support is provided by The Hershey Company, Hershey, PA, USA."

The authors have no financial or other conflict of interest.
